# Dimacrolide Sesquiterpene Pyridine Alkaloids from the Stems of *Tripterygium regelii*

**DOI:** 10.3390/molecules21091146

**Published:** 2016-08-29

**Authors:** Dongsheng Fan, Guo-Yuan Zhu, Ting Li, Zhi-Hong Jiang, Li-Ping Bai

**Affiliations:** State Key Laboratory of Quality Research in Chinese Medicine, Macau Institute for Applied Research in Medicine and Health, Macau University of Science and Technology, Taipa, Macau SAR, China; fandongsheng1985@sina.com (D.F.); gyzhu@must.edu.mo (G.-Y.Z.); tli@must.edu.mo (T.L.)

**Keywords:** *Tripterygium regelii*, dimacrolide sesquiterpene pyridine alkaloids, anti-inflammation

## Abstract

Two new dimacrolide sesquiterpene pyridine alkaloids (DMSPAs), dimacroregelines A (**1**) and B (**2**), were isolated from the stems of *Tripterygium regelii*. The structures of both compounds were characterized by extensive 1D and 2D NMR spectroscopic analyses, as well as HRESIMS data. Compounds **1** and **2** are two rare DMSPAs possessing unique 2-(3′-carboxybutyl)-3-furanoic acid units forming the second macrocyclic ring, representing the first example of DMSPAs bearing an extra furan ring in their second macrocyclic ring system. Compound **2** showed inhibitory effects on the proliferation of human rheumatoid arthritis synovial fibroblast cell (MH7A) at a concentration of 20 μM.

## 1. Introduction

Celastraceae is a large family comprising about 97 genera and 1194 species, which are distributed mainly in the tropics and subtropics. Among them, 14 genera with 192 species are native to China [1]. Some plants from several genera including *Tripterygium*, *Celastrus*, *Euonymus*, and *Maytenus*, are used as folk medicines or traditional Chinese medicines [1,2]. Plants of this family are the richest source of diverse dihydro-β-agarofuran sesquiterpenoids, which have been considered as characteristic metabolites and chemotaxonomic markers of this family [3,4]. This class of sesquiterpenes has attracted researchers’ interest because of a variety of promising bioactivities, such as anti-inflammatory, immunosuppressive, multidrug resistance reversal, cytotoxic, antitumor and anti-HIV activities, etc. [3,4].

Dimacrolide sesquiterpene pyridine alkaloids (DMSPAs) are a structurally unique class of dihydro-β-agarofuran sesquiterpenoids. They were characterized by a polyhydroxylated dihydroagarofuran core and two dicarboxylic acid derivatives linked via four ester bonds forming two macrocyclic systems. DMSPAs are a rare class of compounds naturally occurring in a limited number of plants, such as cathedulin E3 [5,6,7,8], cathedulin E4 [5,6,7,8], cathedulin-K19 [9], and cathedulin-K20 [9] from *Catha edulis* (Forsk), triptonine A [10,11] and triptonine B [10,11] from *Tripterygium hypoglaucum*, and tripterygiumine A [12] from *Tripterygium wilfordii*. Moreover, triptonine B has been reported to inhibit HIV replication with EC_50_ value < 0.10 μg/mL in H9 lymphocytes with a significant therapeutic index (TI) value (TI > 1000) [11].

**Figure 1:**
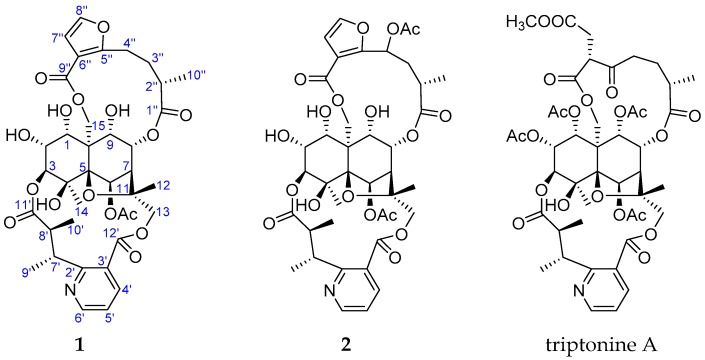
The chemical structures of compounds **1**, **2** and triptonine A.

*Tripterygium regelii* is distributed throughout northeast China, Korea and Japan [13], and has been used as a folk medicine in China to treat rheumatoid arthritis, jaundice, swelling, etc. [14]. A few earlier phytochemical studies on this plant showed the presence of diterpenoids [15], triterpenoids [16,17,18,19,20] and alkaloids [21,22]. Recently, twelve new dihydro-β-agarofuran sesquiterpenoids and three new triterpenoids have been isolated and identified from the stems of *T. regelii* in our laboratory [23,24]. During our ongoing search for secondary metabolites from *T. regelii*, this phytochemical investigation was extended, resulting in the isolation of two new DMSPAs **1** and **2** (Figure 1). Herein, we report the isolation and structural elucidation of both compounds, as well as their inhibitory effect on proliferation of human rheumatoid arthritis synovial fibroblast (MH7A) cells.

## 2. Results and Discussion

**Figure 2:**
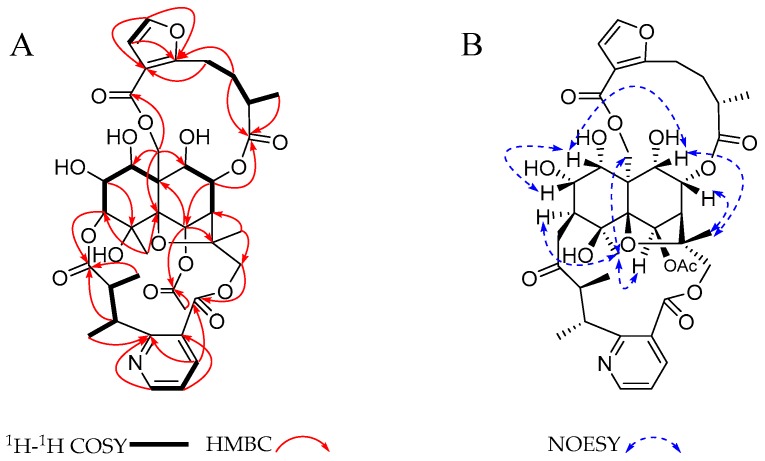
The ^1^H-^1^H COSY, key HMBC and selected NOESY correlations of **1**.

Compound **1** was obtained as a white amorphous powder. The molecular formula of C_38_H_45_NO_16_ was deduced from an [M + H]^+^ ion peak at *m*/*z* 772.2820 (calcd. for C_38_H_46_NO_16_, 772.2811) in the HRESIMS. Its UV spectrum showed absorption bands at λ_max_ 231 and 253 nm, indicating the presence of aromatic rings. In the ^1^H-NMR spectroscopic data of **1** (Table 1), resonances for six oxygenated methines [δ_H_ 6.61 (1H, s, H-6), 5.50 (1H, dd, *J* = 6.0, 3.6 Hz, H-8), 4.75 (1H, d, *J* = 3.0 Hz, H-3), 4.42 (1H, d, *J* = 6.0 Hz, H-9), 4.19 (1H, d, *J* = 3.6 Hz, H-1) and 3.84 (1H, dd, *J* = 3.6, 3.0 Hz, H-2)], one methine [δ_H_ 2.62 (1H, d, *J* = 3.6 Hz, H-7)], two oxygenated methylenes [δ_H_ 5.96 and 3.84 (each 1H, d, *J* = 11.4 Hz, H_2_-13); 5.62 and 4.76 (each 1H, d, *J* =14.4 Hz, H_2_-15)], one hydroxyl proton [4.64 (1H, s), OH-4], two tertiary methyl groups [δ_H_ 1.61 and 1.58 (each 3H, s, H_3_-12 and H_3_-14)] and an acetyl group [δ_H_ 2.12 (3H, s, OAc-6)] indicated the presence of a polyoxygenated dihydro-β-agarofuran sesquiterpene unit [23,25,26,27,28]. An evoninic acid moiety [25,26,27,28,29] was deduced by the signals for a 2,3-disubstituted pyridine [δ_H_ 8.66 (1H, dd, *J* = 4.8, 1.8 Hz, H-6′), 8.16 (1H, dd, *J* = 7.8, 1.8 Hz, H-4′) and 7.39 (1H, dd, *J* = 7.8, 4.8 Hz, H-5′)], two methines [δ_H_ 4.62 (1H, qd, *J* = 6.6, 1.2 Hz, H-7′) and 2.44 (1H, br q, *J* = 6.6 Hz, H-8′)], and two secondary methyl groups [δ_H_ 1.36 and 1.13 (each 3H, d, *J* = 6.6 Hz, H_3_-9′ and H_3_-10′)]. The remaining signals for a 2,3-disubstituted furan [δ_H_ 7.42 (1H, d, *J* = 1.8 Hz, H-8″), 6.73 (1H, d, *J* = 1.8 Hz, H-7″)], a methine [δ_H_ 2.50 (1H, m, H-2″)], two mehylenes [δ_H_ 3.77 (1H, m, H-4″a) and 2.96 (1H, ddd, *J* = 14.4, 7.2, 4.2 Hz, H-4″b); 2.06 and 1.91 (each 1H, m, H_2_-3″)], and one secondary methyl group [δ_H_ 1.14 (3H, d, *J* = 7.2 Hz, H-10″)] were attributed to a 2-(3′-carboxybutyl)-3-furanoic acid unit in the ^1^H-NMR spectrum. The ^13^C-NMR spectroscopic data (Table 1) along with DEPT and HSQC spectra showed the presence of the units mentioned above in **1**. These characteristic NMR data suggested **1** to be a dimacrolide sesquiterpene pyridine alkaloid [5,6,7,8,9,10,11,12]. The ^1^H- and ^13^C-NMR data (Table 1) of **1** were similar to those of triptonine A [10], previously isolated from *T. hypoglaucum*, except for the following two differences: one difference was the different chemical shifts of C-5″, C-6″, C-7″ and C-8″ in **1** from those in triptonine A due to the presence of a furan group in the second macrocyclic ring system in **1**. The 2-(3′-carboxybutyl)-3-furanoic acid unit was disclosed from ^1^H-^1^H COSY correlations of H_3_-10″/H_1_-2″/H_2_-3″/H_2_-4″ and H-7″/H-8″, and the key HMBC correlations from H-3″ and H-10 ″ to C-1″, from H-4″ to C-6″, from H-7″ to C-5″, C-6″, C-8″ and C-9″, and from H-8″ to C-5″, C-6″ and C-7″ (Figure 2A). The linkages of 2-(3′-carboxybutyl)-3-furanoic acid unit and dihydro-β-agarofuran sesquiterpene core were via C-8−O−C-1″ and C-15−O−C-9″, as deduced from the key HMBC correlations from H-8 (δ_H_ 5.50) and H_2_-15 (δ_H_ 5.62 and 4.76) to the carbonyl carbons (δ_C_ 177.1, C-1″) and (δ_C_ 166.2, C-9″), respectively. The other key difference was the absence of three acetyl groups and the upfield shifts of H-1 (δ_H_ 4.19), H-2 (δ_H_ 3.84) and H-9 (δ_H_ 4.42) relative to those (δ_H_ 5.46, 5.15, and 5.25, respectively) in triptonine A, which suggested deacetylation of the C-1, C-2 and C-9 in **1**. Therefore, the proposed planar structure of **1** was established and further confirmed by the ^1^H-^1^H COSY and HMBC analyses (Figure 2A).

The relative configuration of the dihydro-β-agarofuran sesquiterpene core in **1** was assigned by the NOESY correlations (Figure 2B) and coupling constant. The NOE correlations of H-1/H-9, H-9/H_3_-12 and H_3_-12/H-8 suggested that these protons were on the same face. NOE effects of H_3_-14/H-3, H_3_-14/H-6 and H_3_-14/H_2_-15 were observed, showing that these protons were on the other face. The small coupling constant of *J*_1,2_ = 3.6 Hz between H-1 and H-2 indicated that the H-2 was equatorial. These data revealed that the relative configuration of the dihydro-β-agarofuran sesquiterpene core in **1** was identical to that of triptonine A [10] and the related compounds [12,27,28]. The relative configurations of the groups at two macrocyclic rings were determined by comparison of the NMR spectroscopic data with those of triptonine A [10], which was established by X-ray crystallography. As the ^1^H and ^13^C data of **1** were closely similar to those of triptonine A except for positions C-5″, C-6″, C-7″ and C-8″, it could indicate the same relative configurations at C-7′, C-8′ and C-2″ in the macrocyclic rings. Therefore, compound **1** was identified as shown in Figure 1, and given a trivial name of dimacroregeline A. 

Compound **2** was isolated as a white amorphous powder. The molecular formula was determined as C_40_C_47_NO_18_ on the basis of a protonated molecule at *m*/*z* 830.2876 [M + H]^+^ (calcd for C_40_C_48_NO_18_, 830.2866), which was 58.0056 mass units more than that of **1** in the HRESIMS. The ^1^H and ^13^C-NMR spectroscopic data of **2** (Table 1) were closely similar to those of **1** except for the absence of C-4″ methylene group and the presence of an additional oxygenated methine (δ_H_ 6.89, δ_C_ 68.0, CH-4″) and an extra acetyl group (δ_H_ 1.96, δ_C_ 171.6 and 20.9). The methylene group (δ_H_ 3.77 and 2.96) at C-4″ in **1** was displaced by the oxygenated methine (δ_H_ 6.89, H-4″) in **2**, supported by the observation of HMBC correlation from the oxygenated methine (δ_H_ 6.89) to the carbon signal (δ_C_ 37.9) at C-3″ in **2**. The acetyl group (δ_H_ 1.96, δ_C_ 171.6 and 20.9) was allocated to C-4″ in **2**, as evidenced from the HMBC correlation from H-4″ signal (δ_H_ 6.89) to the carbonyl carbon (δ_C_ 171.6) of the acetyl group. Thus, compound **2** was elucidated as shown in Figure 1, and named dimacroregeline B.

So far, only seven dimacrolide sesquiterpene pyridine alkaloids [5,6,7,8,9,10,11,12] have been isolated from plants. Compounds **1** and **2** represent the first example of dimacrolide sesquiterpene pyridine alkaloids bearing an extra furan ring in their second macrocyclic ring system. The HRMS, UV, NMR and CD spectra of compounds **1** and **2** were shown in the supplementary materials (Figures S1–S18).

**Scheme 1:**
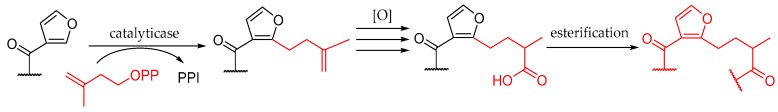
Plausible biosynthetic pathway for 2-(3′-carboxybutyl)-3-furanoic acid unit.

Over the past several decades, many monomacrolide sesquiterpene pyridine alkaloids have been identified from the plants in the family Celastraceae, especially *Tripterygium* [3,4], the proposed biosynthetic pathways for dihydro-β-agarofuran sesquiterpene unit and pyridine dicarboxylic acid moiety have been disclosed [9,30,31]. The biosynthetic pathway of 2-(3′-carboxybutyl)-3-furanoic acid unit was proposed as shown in Scheme 1, since this unit was reported in nature for the first time. The 3-furanoic acid residue is regarded as its biosynthetic precursor, which could generate this unit via prenylation under catalysis in plants [32].

It is well known that activated synovial fibroblasts play a crucial role in the pathogenesis of rheumatoid arthritis (RA), which could result in the cartilage destruction and synovial inflammation [33,34,35]. The inhibition of the synovial fibroblast proliferation is considered as a promising treatment for RA. Thus, compounds **1** and **2** were evaluated for anti-proliferative activity against human rheumatoid arthritis synovial fibroblast cell line (MH7A). As the result (Table 2), compound **2** inhibited proliferation of MH7A cells (*p* < 0.05) by 13.3% at the concentration of 20 μM compared with the vehicle control.

## 3. Materials and Methods

### 3.1. General Procedures

Optical rotations and ultraviolet (UV) spectra were recorded on a Rudolph Research Analytical Autopol I automatic polarimeter (Rudolph Research Analytical, Hackettstown, NJ, USA) and a DU^®^ 800 spectrophotometer (Beckman Coulter, Fullerton, CA, USA), respectively. Circular dichroism spectra were measured on a Jasco J1500 CD spectrometer (Jasco Corperation, Tokyo, Japan). Nuclear magnetic resonance (NMR) spectra were acquired with an Ascent 600 NMR spectrometer at 600 MHz for ^1^H-NMR and 150 MHz for ^13^C-NMR (Bruker, Zurich, Switzerland). The samples dissolved in CD_3_OD with residual solvent as an internal reference. HRMS spectra were performed on a 6230 electrospray ionization (ESI) time-of-flight (TOF) mass spectrometer (Agilent, Santa Clara, CA, USA) in the positive ion mode. Medium pressure liquid chromatography (MPLC) was carried out on a Sepacore Flash Chromatography System (Buchi, Flawil, Switzerland) using a flash column (460 × 36 mm, i.d., Buchi) packed with Bondapak Waters ODS (40–63 μm, Waters, Milford, MA, USA). Preparative HPLC was conducted on a Waters liquid chromatography system coupled with 1525 Binary HPLC Pump and 2489 UV/Visible detector using a Waters Xbridge Prep C_8_ column (10 × 250 mm, 5 μm). Semi-preparative HPLC was performed on an Agilent 1100 liquid chromatography system equipped with a quaternary pump and a diode array detector (DAD) using a Waters Xbridge Prep C_18_ column (10 × 250 mm, 5 μm). Silica gel (40–60 μm, Grace, Columbia, MD, USA) was used for column chromatography. Thin layer chromatography (TLC) was performed on precoated silica gel 60 F_254_ plates and TLC silica gel 60 RP-18 F_254S_ plates (200 µm thick, Merck KGaA, Darmstadt, Germany).

### 3.2. Plant Material

The stems of *T. regelii* were collected from Changbai Mountain in Jilin Province, China, in October 2012, and authenticated by Dr. Liang Xu, School of Pharmacy, Liaoning University of Traditional Chinese Medicine, Dalian, China. A voucher specimen (No. MUST-TR201210) has been deposited at State Key Laboratory of Quality Research in Chinese Medicine, Macau University of Science and Technology, Macau, China.

### 3.3. Extraction and Isolation

The air-dried and crushed stems of *T. regelii* (8.0 kg) were extracted three times with MeOH (64 L) under ultrasound at room temperature for 1 h. A dark brown residue was obtained after removing the solvent under reduced pressure, which was suspended in H_2_O, and then partitioned with *n*-hexane, EtOAc and *n*-butanol. The EtOAc-soluble extract (150.0 g) was chromatographed over a silica gel column and eluted with petroleum ether–acetone (100:0–35:65, *v*/*v*) to give thirteen fractions (Fr. 1–Fr.13). The Fr.12 (9.0 g) was separated by MPLC with a gradient of MeOH–H_2_O (5:95–100:0, 50 mL/min) to afford six fractions (Fr.12-1–Fr.12-6). The Fr.12-5 showed the alkaloid-positive test after spraying with Dragendorff’s reagent. Then, Fr.12-5 (350 mg) was separated by preparative HPLC using MeCN–H_2_O (42:58, *v*/*v*) as mobile phase to give eleven fractions (Fr.12-5-1–Fr.12-5-11). Fr.12-5-2 (50 mg) was isolated by semi-preparative HPLC with a MeCN–H_2_O (39:61, *v*/*v*) solvent system to afford compound **2** (1.22 mg) and subfraction 12-5-2-3 (5 mg). Then, the subfraction 12-5-2-3 was further purified by semi-preparative HPLC using MeOH–H_2_O (40:60, *v*/*v*) as mobile phase to yield compound **1** (1.01 mg).

*Dimacroregeline A* (**1**) White amorphous powder; ${\left[\alpha \right]}_{D}^{25}$ −19.8 (*c* 0.25, MeOH); UV (MeOH) λ_max_ (log ε) 231 (3.95), 253 (3.84) nm; CD (*c* 6.5 × 10^−4^ mol/L, MeOH) λ_max_ (Δε) 200 (+14.60), 216 (sh) (+3.96), 241 (−3.96) nm; ^1^H-NMR (CD_3_OD) and ^13^C-NMR (CD_3_OD) data, see Table 1; HRESIMS *m*/*z* 772.2820 [M + H]^+^ (calcd for C_38_H_46_NO_16_, 772.2811), HRESIMS *m*/*z* 794.2634 [M + Na]^+^ (calcd. for C_38_H_45_NO_16_Na, 794.2631).

*Dimacroregeline B* (**2**) White amorphous powder; ${\left[\alpha \right]}_{D}^{25}$ +4.0 (*c* 0.50, MeOH); UV (MeOH) λ_max_ (log ε) 228 (3.98), 260 (3.65) nm; CD (*c* 6.0 × 10^−4^ mol/L, MeOH) λ_max_ (Δε) 233 (+22.64), 245 (+17.36), 276 (−2.90) nm; ^1^H-NMR (CD_3_OD) and ^13^C-NMR (CD_3_OD) data, see Table 1; HRESIMS *m*/*z* 830.2876 [M + H]^+^ (calcd for C_40_H_48_NO_18_, 830.2866), HRESIMS *m*/*z* 852.2695 [M + Na]^+^ (calcd. for C_40_H_47_NO_18_Na, 852.2685).

### 3.4. Inhibition of Proliferation on MH7A Human Synovial Cells

MH7A cells were obtained from American Type Culture Collection (ATCC, Manassas, VA, USA). Cells were cultured in a Dulbecco’s modified Eagle medium (DMEM) supplemented with 10% fetal bovine serum (FBS, Invitrogen, Carlsbad, CA, USA) and 1% penicillin-streptomycin (Sigma, St. Louis, MO, USA) at 37 °C in humidified atmosphere containing 5% CO_2_. MTT [3-(4,5-dimethylthiazol-2-yl)-2,5-diphenyl tetrazolium bromide] was employed to determine the cell viability as described previously [36,37]. Briefly, 5 × 10^3^ cells/well MH7A cells (total 100 µL) were cultured in triplicate in a 96-well plate with or without 20 μM compounds for 48 h incubation at 37 °C in humidified atmosphere containing 5% CO_2_. MTT (5 mg/mL, 10 µL) was added into each well and incubated for 4 h before terminating the culture. The supernatant was gently removed, and the formazan in each well was dissolved in the lysing solvent (10% sodium dodecyl sulfate (SDS), 50% *N*,*N*-dimethylformamide, pH 7.2). Absorbance at 570 nm was determined using a microplate reader (Infinite 200 PRO, Tecan, Männedorf, Switzerland) from each well on the next day. The percentage of cell viability was calculated using the following formula: Cell viability (%) = (Absorbance of treated with compounds/Absorbance of treated with control vehicle) × 100. Data reported represent three independent experiments. Data are expressed as means ± SEM. One-way ANOVA was used to determine the significance of difference. A value of *p* < 0.05 was considered statistically significant.

## Figures and Tables

**Table 1:** ^1^H- (600 MHz) and ^13^C- (150 MHz) NMR spectroscopic data for **1** and **2** in CD_3_OD.

Position	1		2	
	δ_H_ (*J* in Hz)	δ_C_, Type	δ_H_ (*J* in Hz)	δ_C_, Type
1	4.19, d (3.6)	75.4, CH	4.24, d (3.6)	74.9, CH
2	3.84, dd (3.6, 3.0)	73.0, CH	3.83, dd (3.6, 2.4)	73.0, CH
3	4.75, d, (3.0) ^a^	80.4, CH	4.79, d (2.4)	80.2, CH
4		72.2, C		72.3, C
5		95.3, C		94.9, C
6	6.61, s	76.6, CH	6.20, s	78.5, CH
7	2.62, d (3.6)	51.1, CH	2.94, d (4.2)	50.0, CH
8	5.50, dd (6.0, 3.6)	72.9, CH	5.44, dd (6.0, 4.2)	73.4, CH
9	4.42, d (6.0)	73.6, CH	4.43, d (6.0)	73.7, CH
10		54.8, C		55.2, C
11		85.1, C		84.7, C
12	1.61, s	18.4, CH_3_	1.59, s	18.2, CH_3_
13 a	5.96, d (11.4)	71.6, CH_2_	5.78, d (11.4)	71.4, CH_2_
13 b	3.84, d (11.4) ^a^		3.81, d (11.4)	
14	1.58, s	24.1, CH_3_	1.77, d (0.6)	24.6, CH_3_
15 a	5.62, d (14.4)	63.2, CH_2_	5.21, d (14.4)	64.1, CH_2_
15 b	4.76, d (14.4) ^a^		5.01, d (14.4)	
2′		165.7, C		165.3, C
3′		127.2, C		127.4, C
4′	8.16, dd (7.8, 1.8)	139.2, CH	8.13, dd (7.8, 1.8)	139.0, CH
5′	7.39, dd (7.8, 4.8)	122.8, CH	7.39, dd (7.8, 4.8)	122.8, CH
6′	8.66, dd (4.8, 1.8)	152.4, CH	8.66, dd (4.8, 1.8)	152.4, CH
7′	4.62, qd (6.6, 1.2) ^a^	37.6, CH	4.57, qd (6.6, 1.2) ^a^	37.7, CH
8′	2.44, br q (6.6)	46.3, CH	2.46, qd (6.6, 1.2)	46.2, CH
9′	1.36, d (6.6)	12.0, CH_3_	1.36, d (6.6)	12.2, CH_3_
10′	1.13, d (6.6)	9.8, CH_3_	1.16, d (6.6)	9.9, CH_3_
11′		175.9, C		175.9, C
12′		170.1, C		170.0, C
1″		177.1, C		176.5, C
2″	2.50, m	38.0, CH	2.16, m	35.5, CH
3″ a	2.06, m	33.2, CH_2_	2.36, td (12.6, 4.8)	37.9, CH_2_
3″ b	1.91, m		2.25, td (12.6, 1.8)	
4″ a	3.77, m	26.8, CH_2_	6.89, dd (12.6, 4.8)	68.0, CH
4″ b	2.96, ddd (14.4, 7.2, 4.2)			
5″		161.3, C		154.3, C
6″		116.0, C		119.9, C
7″	6.73, d (1.8)	113.4, CH	6.80, d (1.8)	113.5, CH
8″	7.42, d (1.8)	142.3, CH	7.58, d (1.8)	144.1, CH
9″		166.2, C		165.6, C
10″	1.14, d (7.2)	17.4, CH_3_	1.10, d (6.6)	17.7, CH_3_
OH-4	4.64, s ^a^		4.57, s ^a^	
OAc-6	2.12, s	21.5, CH_3_	2.09, s	21.7, CH_3_
		171.3, C		171.9, C
OAc-4″			1.96, s	20.9, CH_3_
				171.6, C

^a^ The overlapped signals were assigned from ^1^H-^1^H COSY, HSQC, and HMBC spectra.

**Table 2:** Effect of compounds **1** and **2** on the viability of MH7A cell.

Compounds ^#^	Cell Viability (%) (*n* = 3)	Inhibition Rate (%)
Vehicle control	100 ± 5.9	0
**1**	94.6 ± 5.4	5.4 ± 5.4
**2**	86.7 ± 5.2 *	13.3 ± 5.2 *

* There was a significant difference (*p* < 0.05) between the group treated with compound **2** and the control group treated with the vehicle; ^#^ Compounds **1** and **2** were tested at the concentration of 20 µM.

## Supplementary Materials

Supplementary materials can be accessed at: http://www.mdpi.com/1420-3049/21/9/1146/s1.
