# Supplementary material for: Dimacrolide Sesquiterpene Pyridine Alkaloids from the Stems of Tripterygium regelii
Source: Molecules. 2016 Aug 29;21(9):1146. doi: 10.3390/molecules21091146 (PMC6273108; doi:10.3390/molecules21091146)
Supplement: Supplementary file 1 [file molecules-21-01146-s001.pdf]

Supplementary Materials: Dimacrolide Sesquiterpene Pyridine Alkaloids from the Stems of *Tripterygium regelii*

Dongsheng Fan, Guo-Yuan Zhu, Ting Li, Zhi-Hong Jiang and Li-Ping Bai

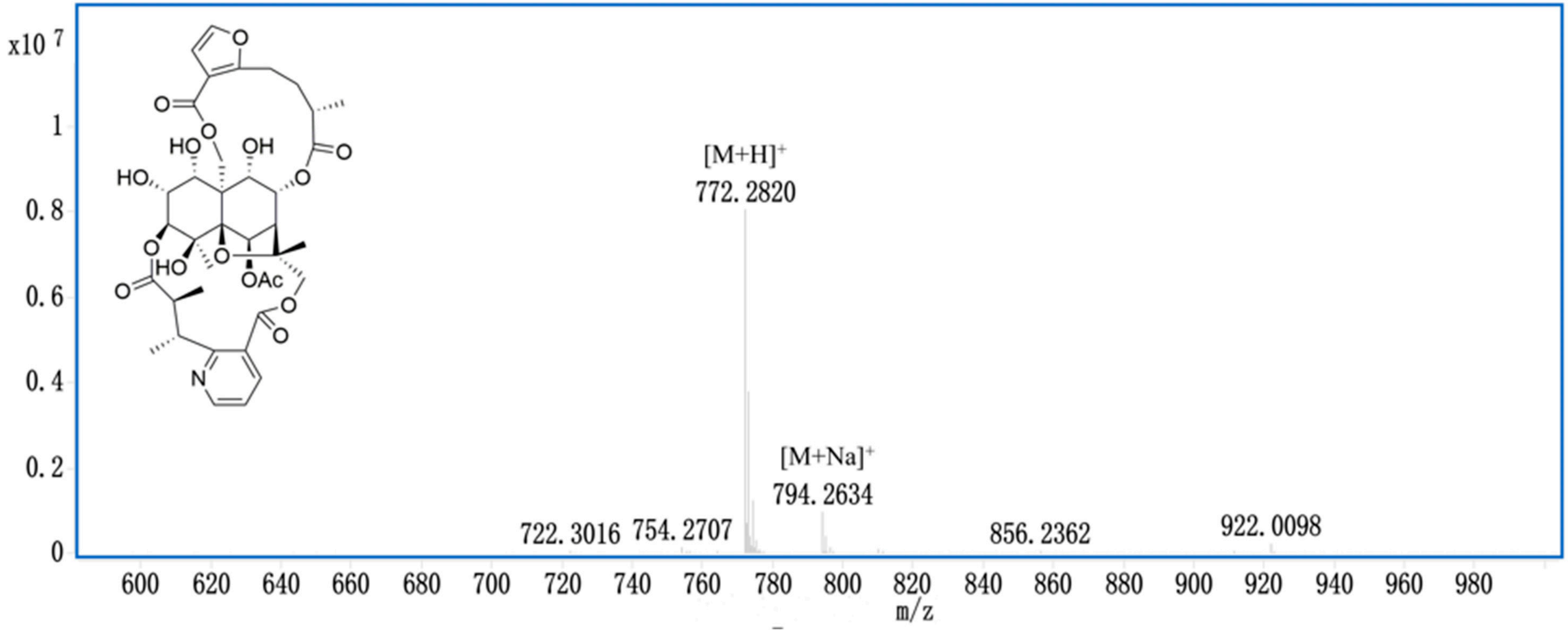

Figure S1. HRESIMS spectrum of compound **1**.

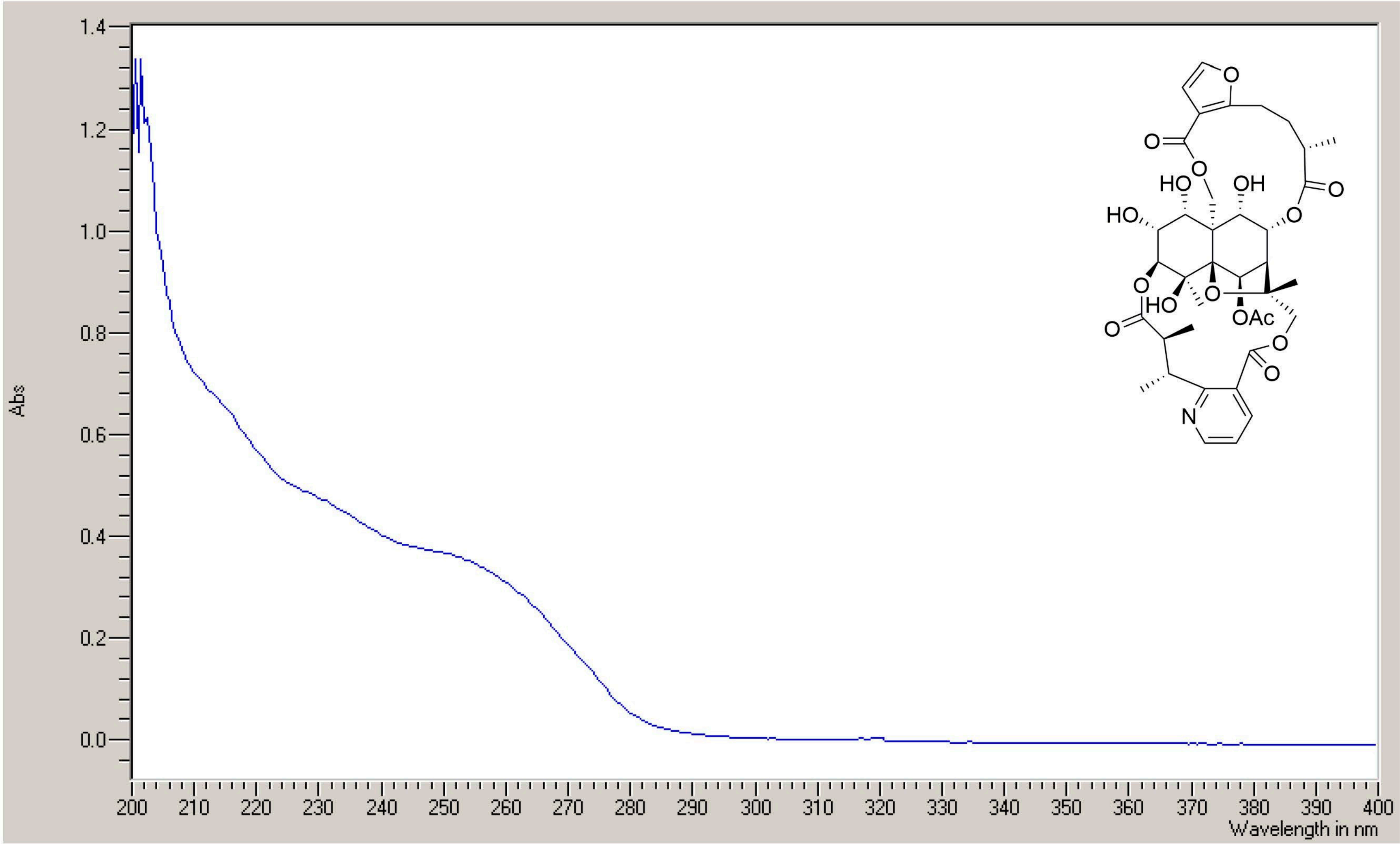

Figure S2. UV spectrum of compound **1** in CH_3OH.

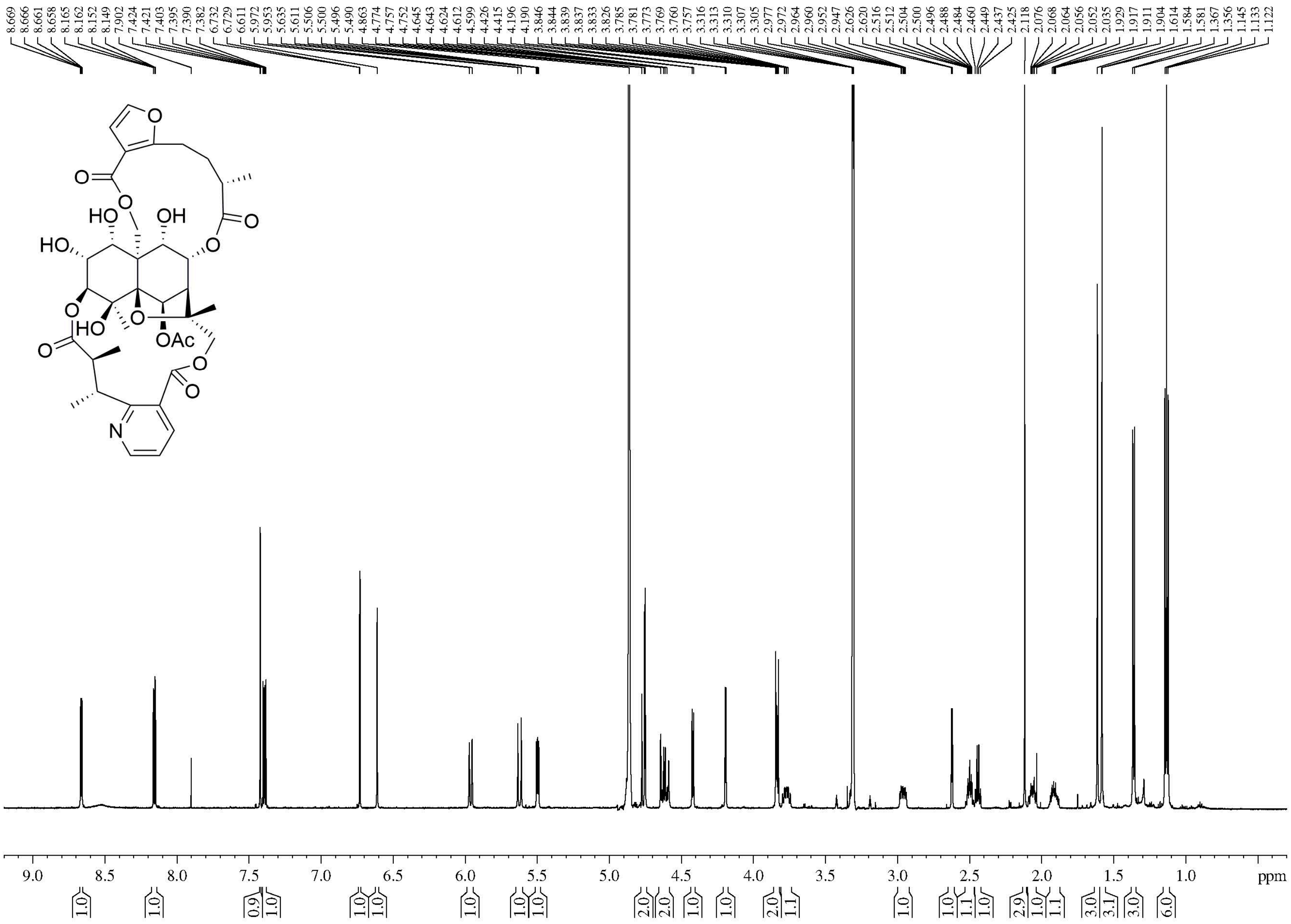

Figure S3. ^{1}H-NMR spectrum of compound **1** in CD_3OD.

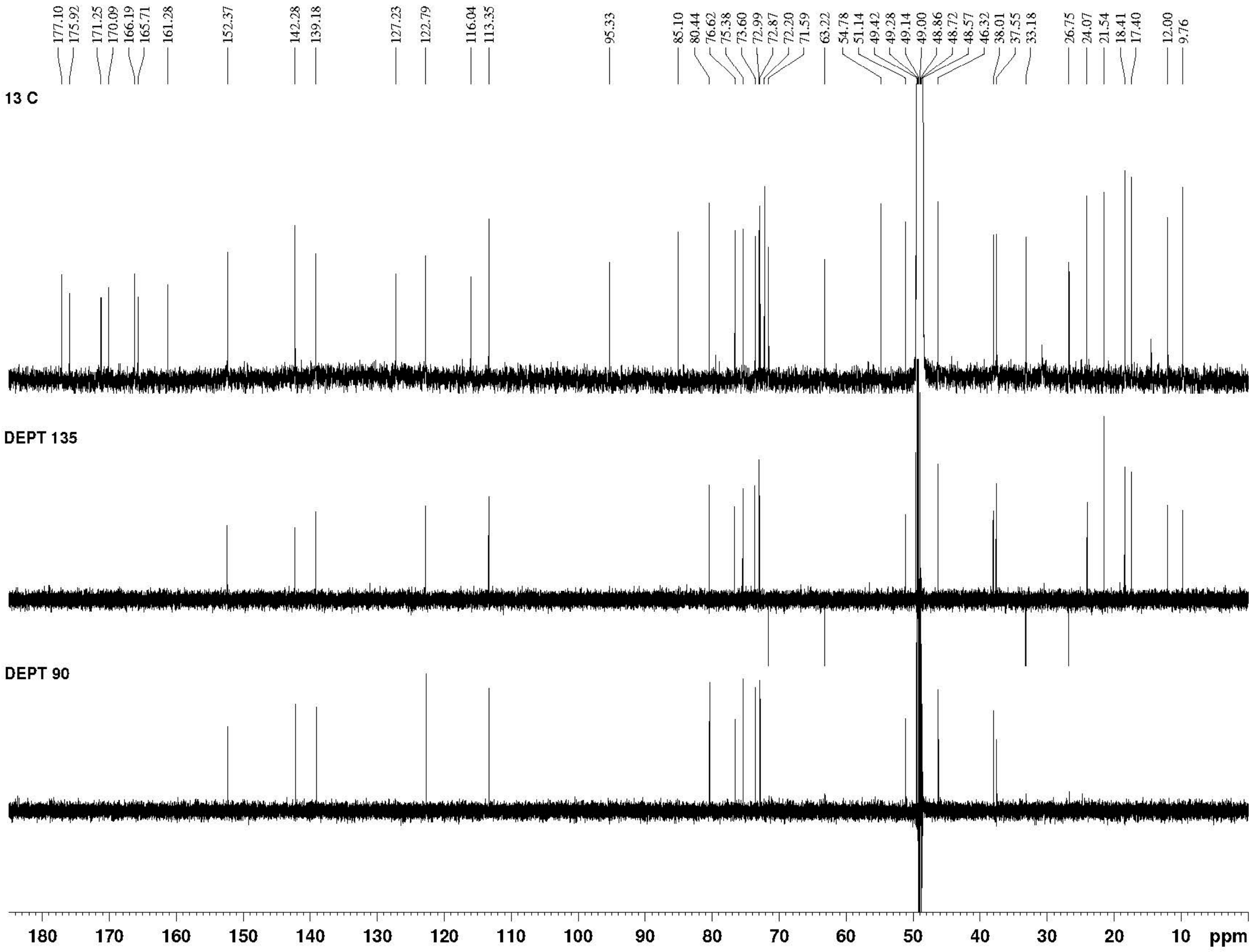

Figure S4. ^{13}C and DEPT NMR spectra of compound **1** in CD_3OD.

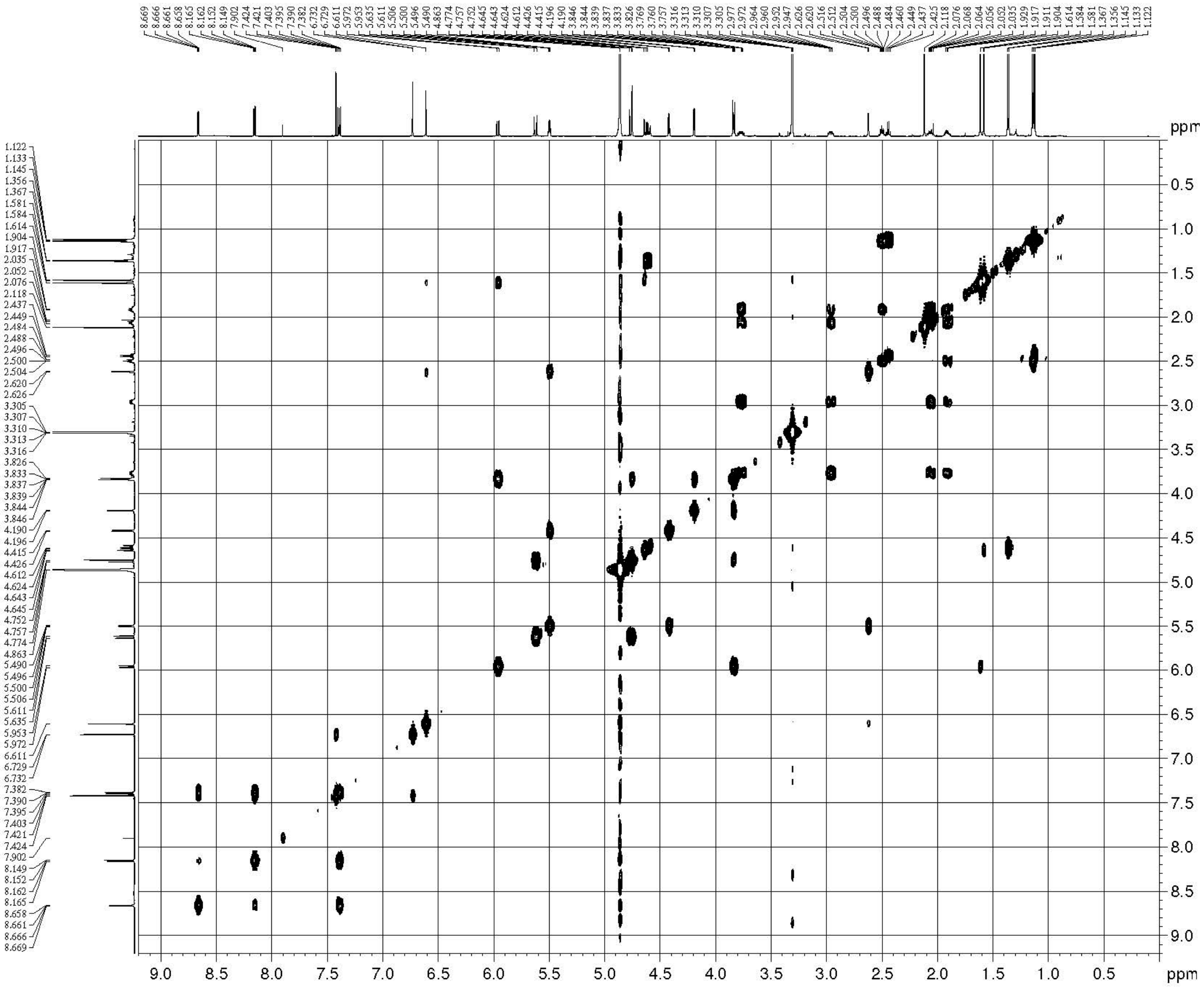

Figure S5. ¹H-¹H COSY spectrum of compound **1** in CD_3OD.

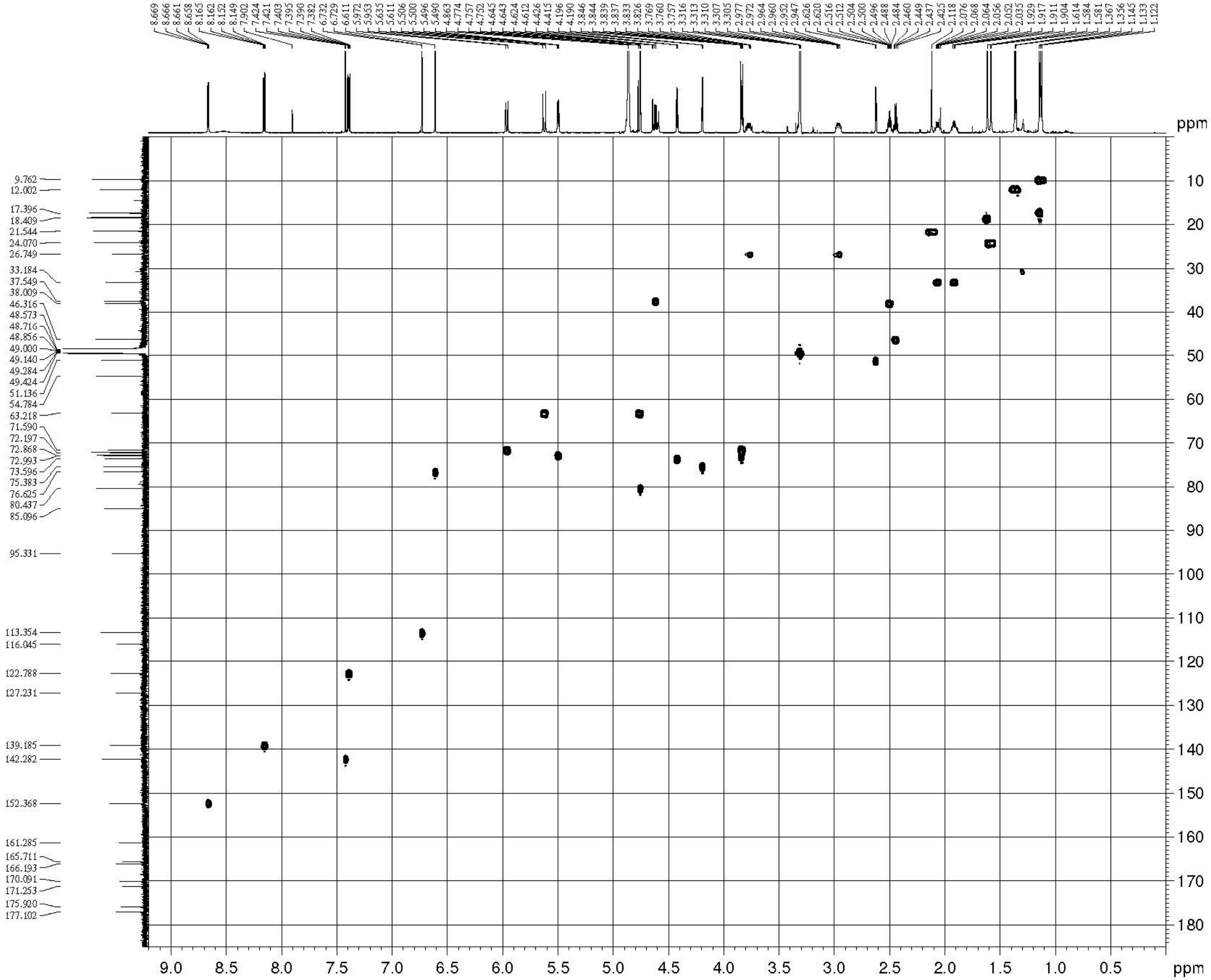

Figure S6. HSQC spectrum of compound **1** in CD_3OD.

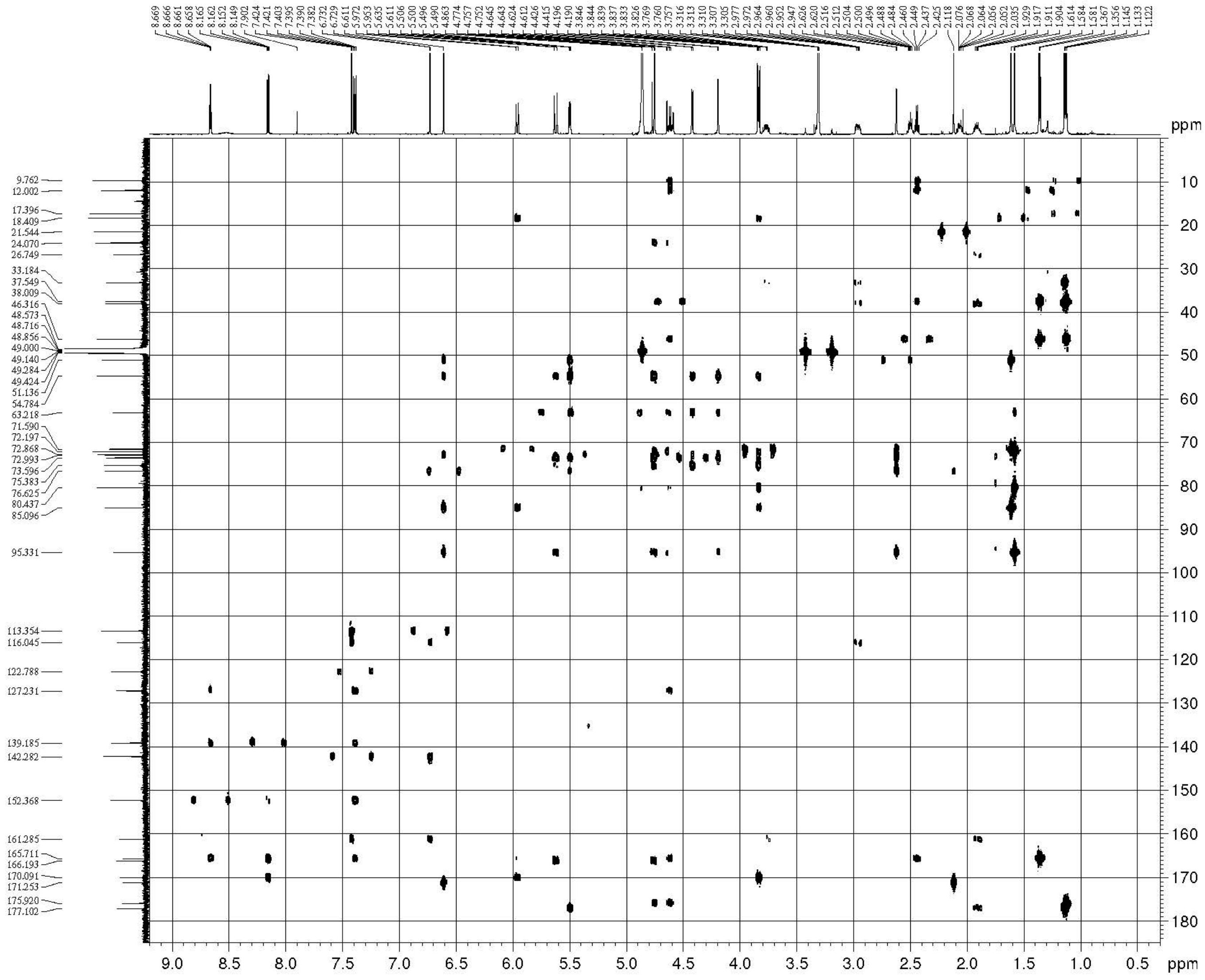

Figure S7. HMBC spectrum of compound **1** in CD_3OD.

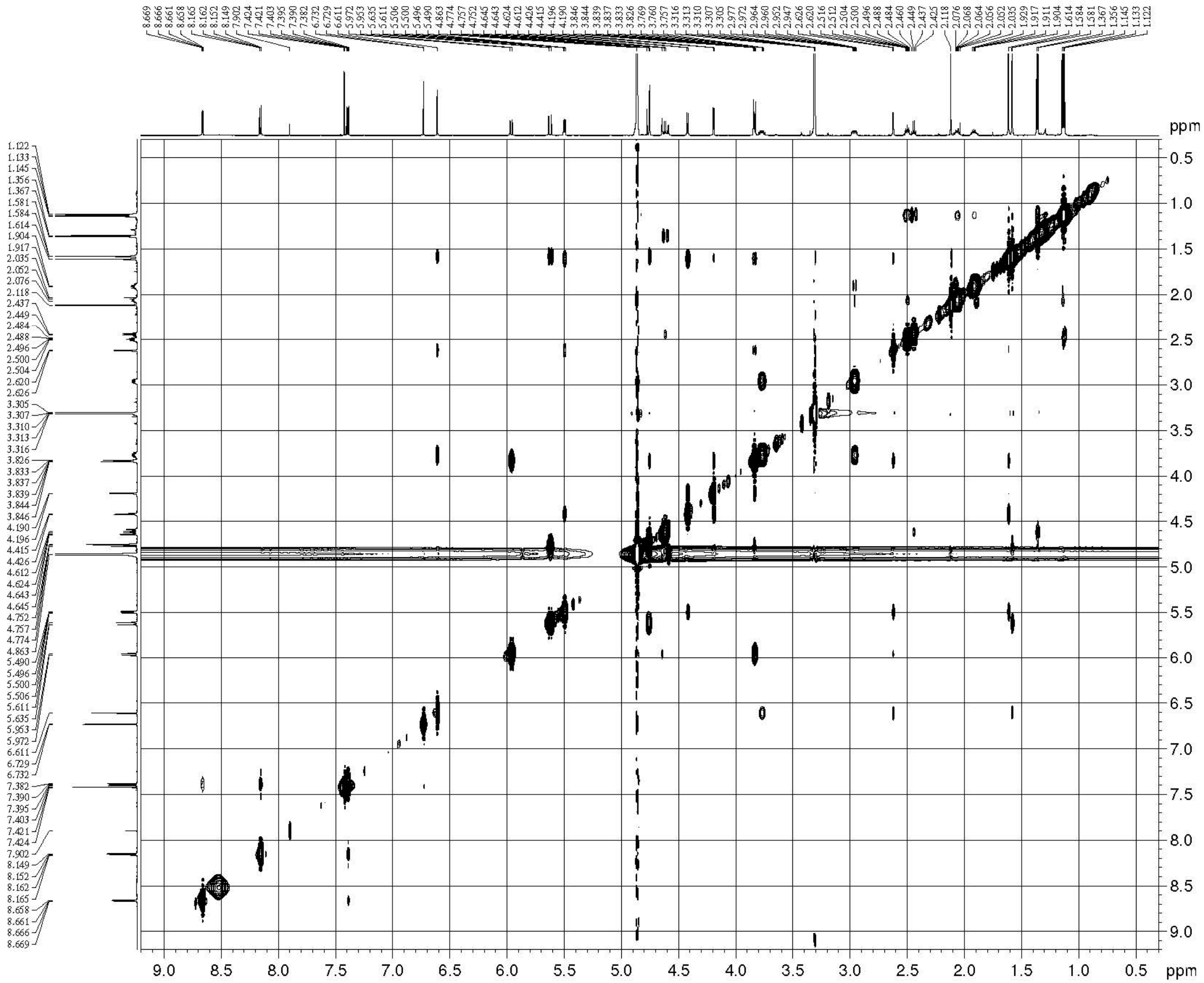

Figure S8. NOESY spectrum of compound **1** in CD_3OD.

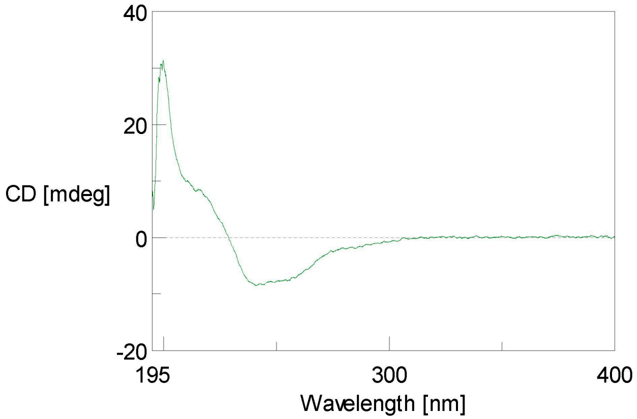

Figure S9. CD spectrum of compound **1** in MeOH.

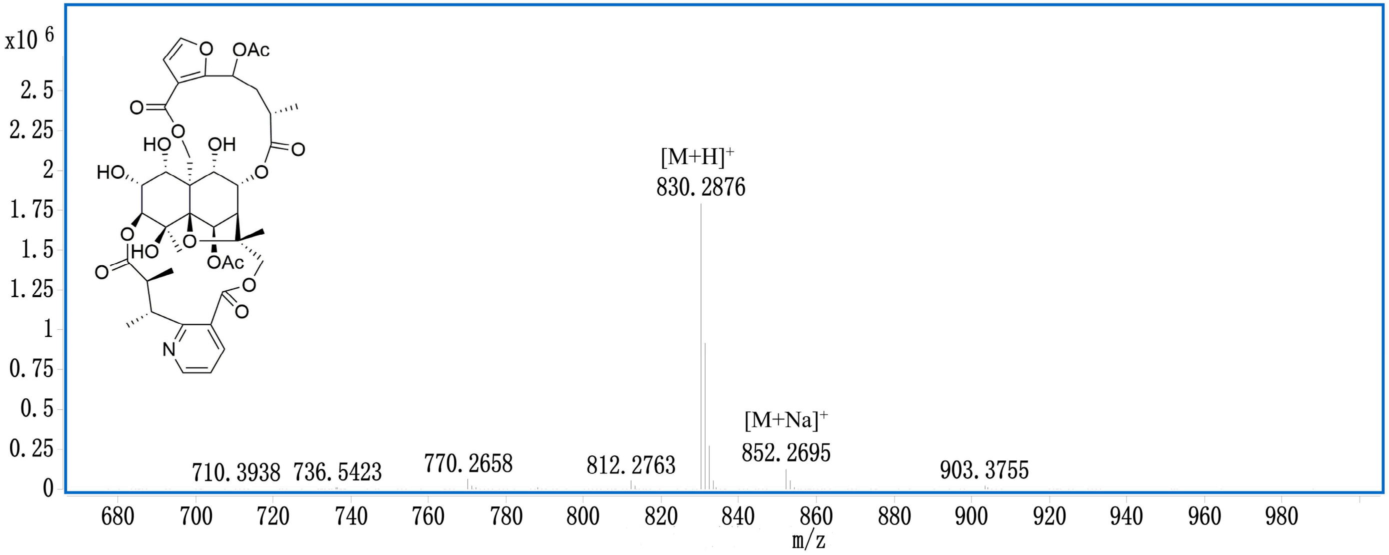

Figure S10. HRESIMS spectrum of compound **2**.

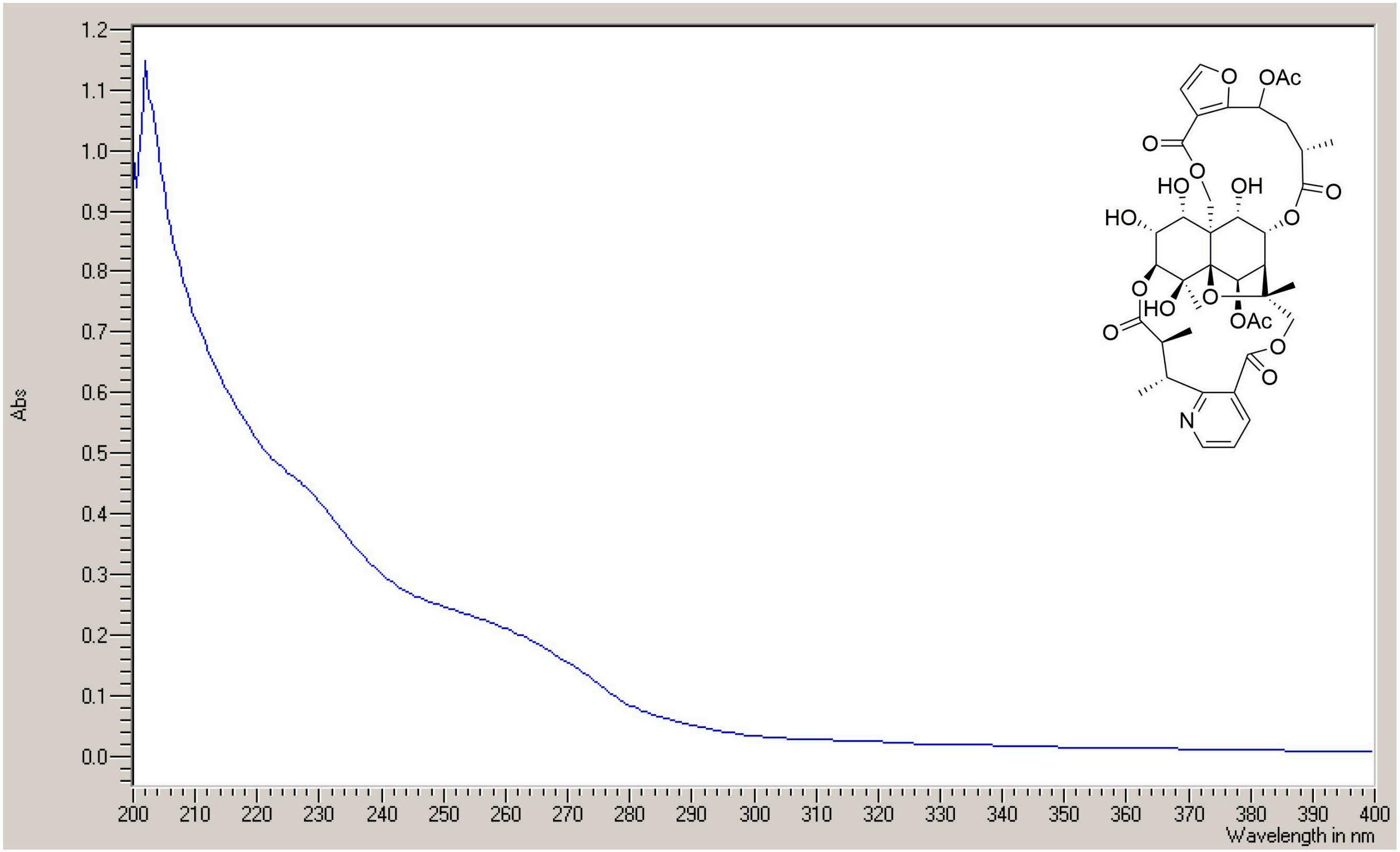

Figure S11. UV spectrum of compound **2** in CH_3OH.

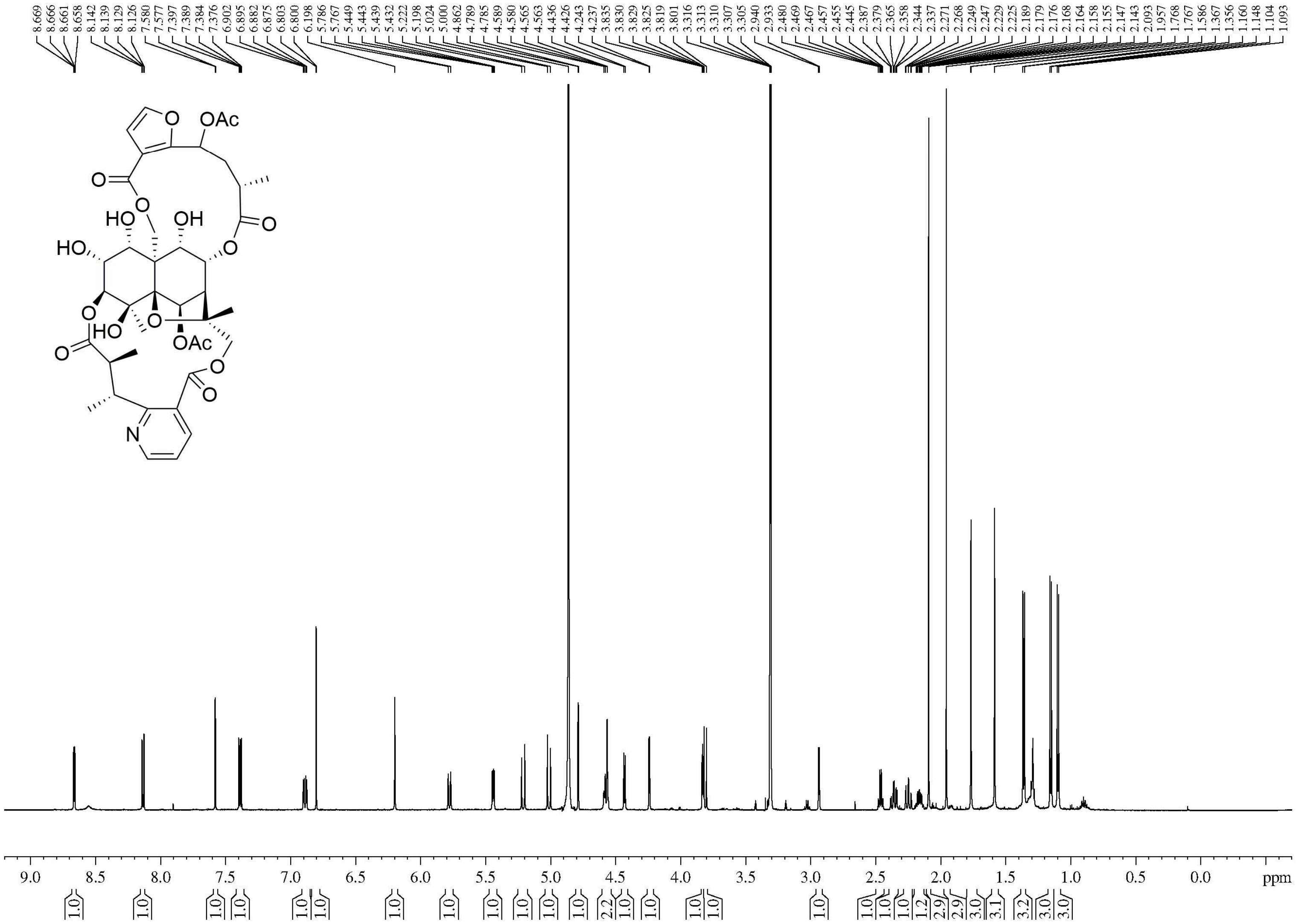

Figure S12. ^{1}H-NMR spectrum of compound **2** in CD_3OD.

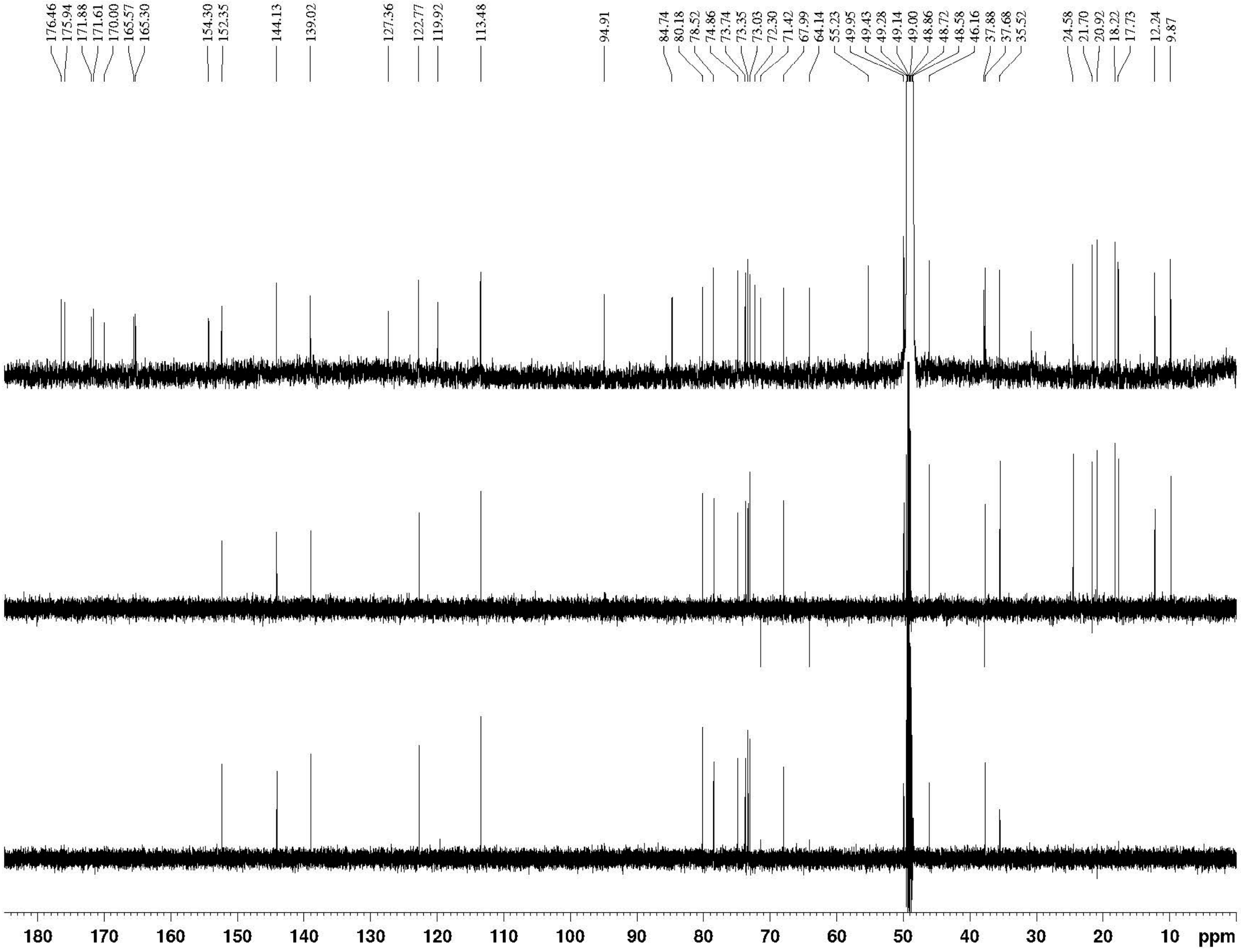

Figure S13. ^{13}C and DEPT NMR spectra of compound **2** in CD_3OD.

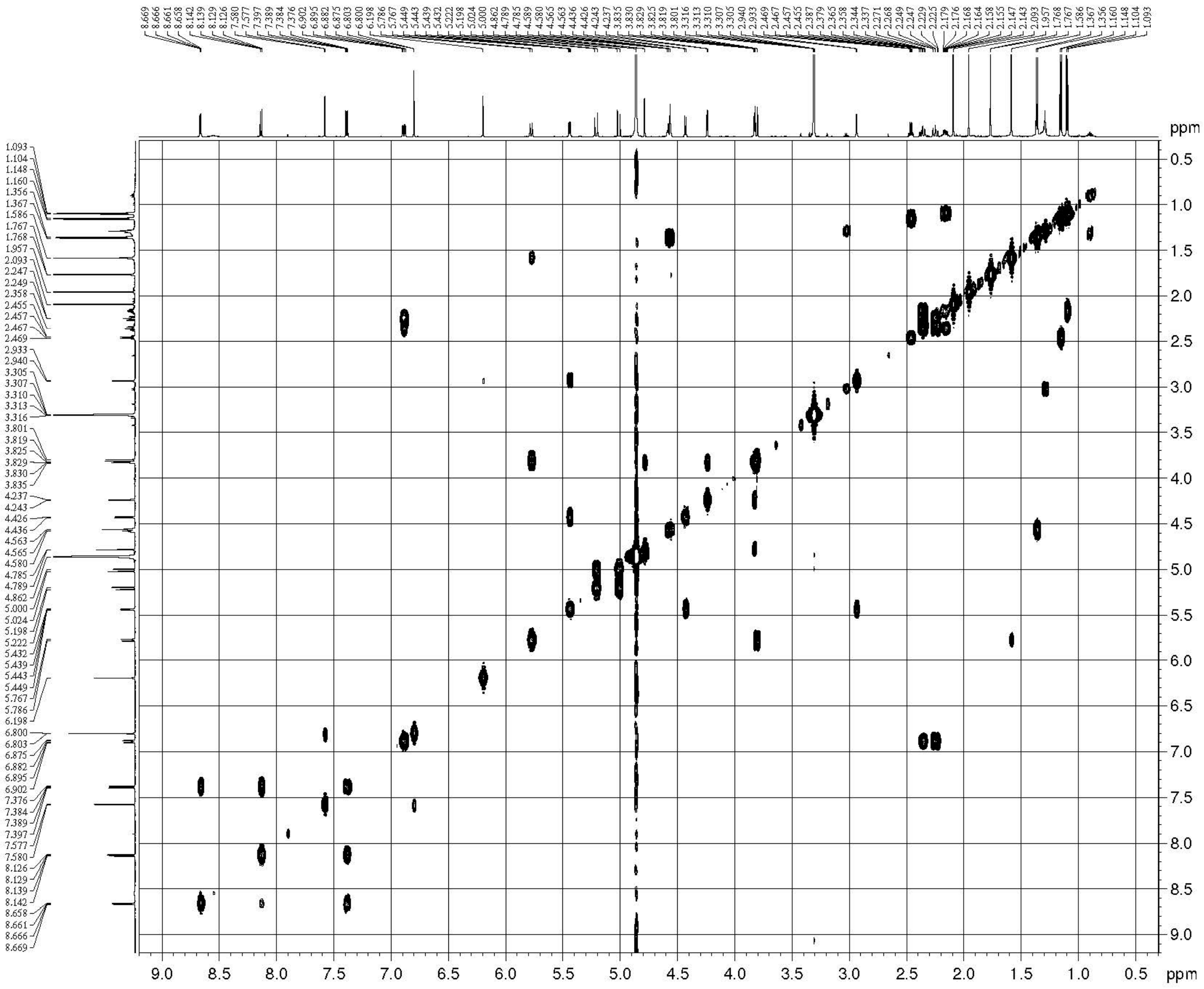

Figure S14. ^1H-^1H COSY spectrum of compound **2** in CD_3OD.

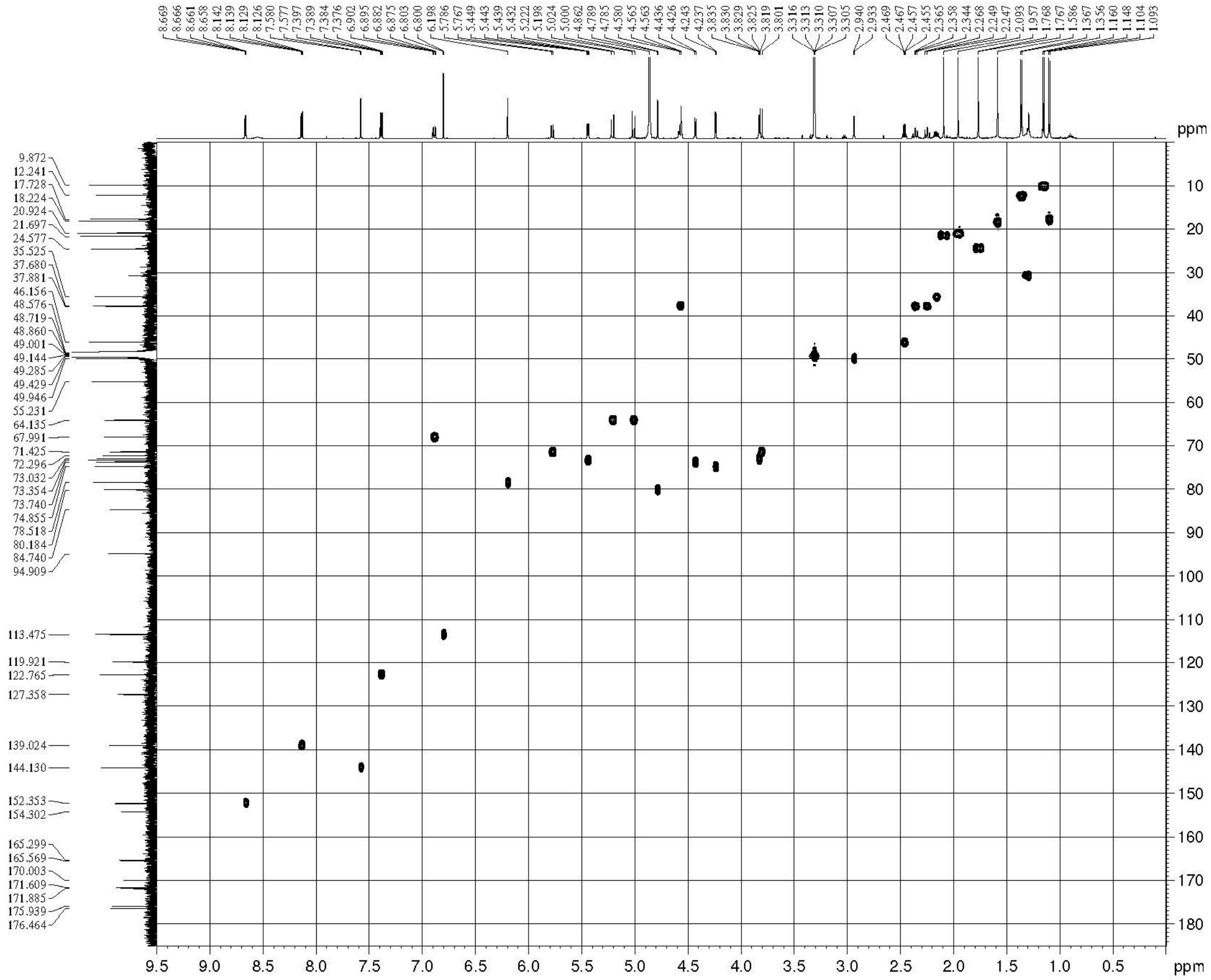

Figure S15. HSQC spectrum of compound **2** in CD_3OD.

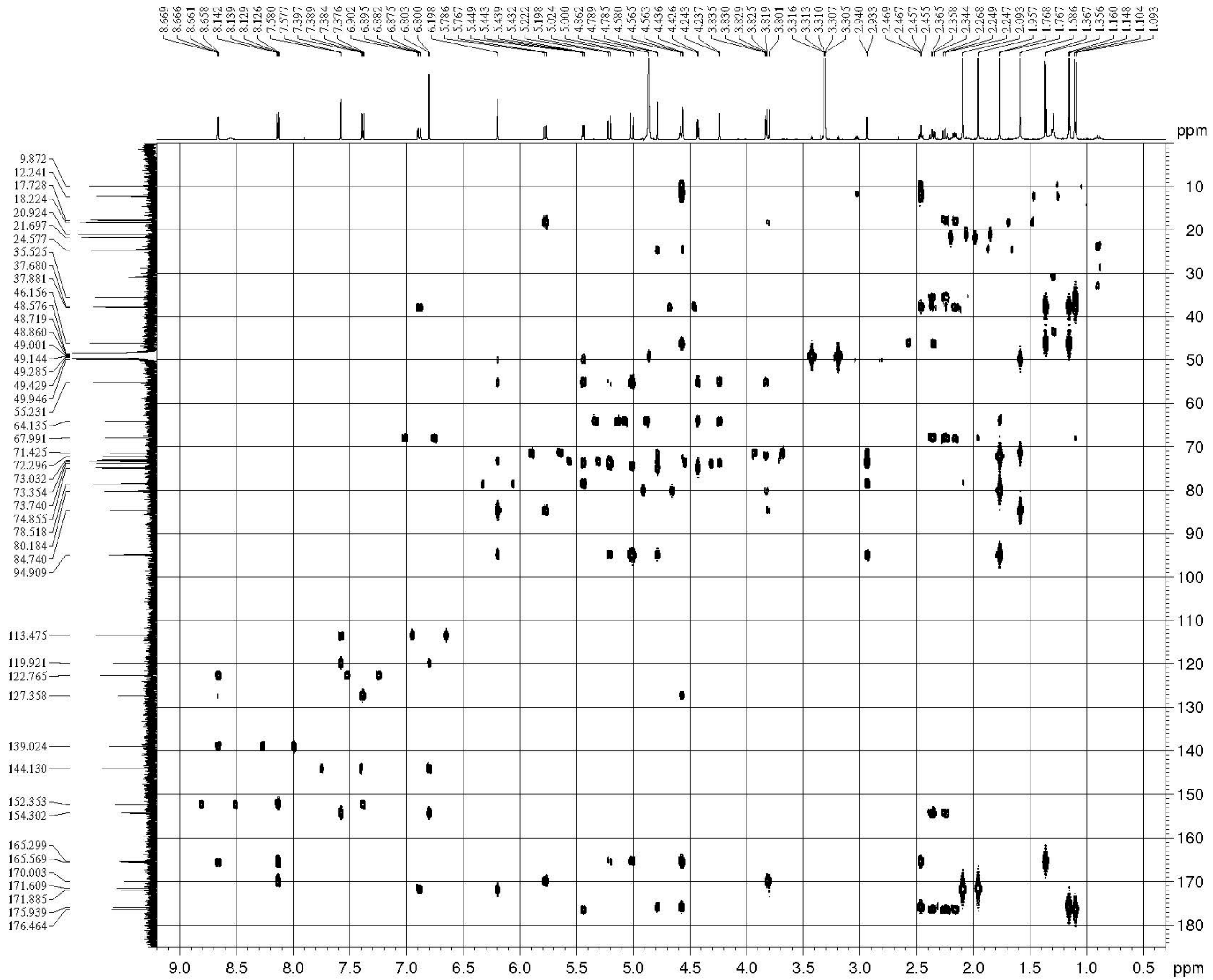

Figure S16. HMBC spectrum of compound **2** in CD_3OD.

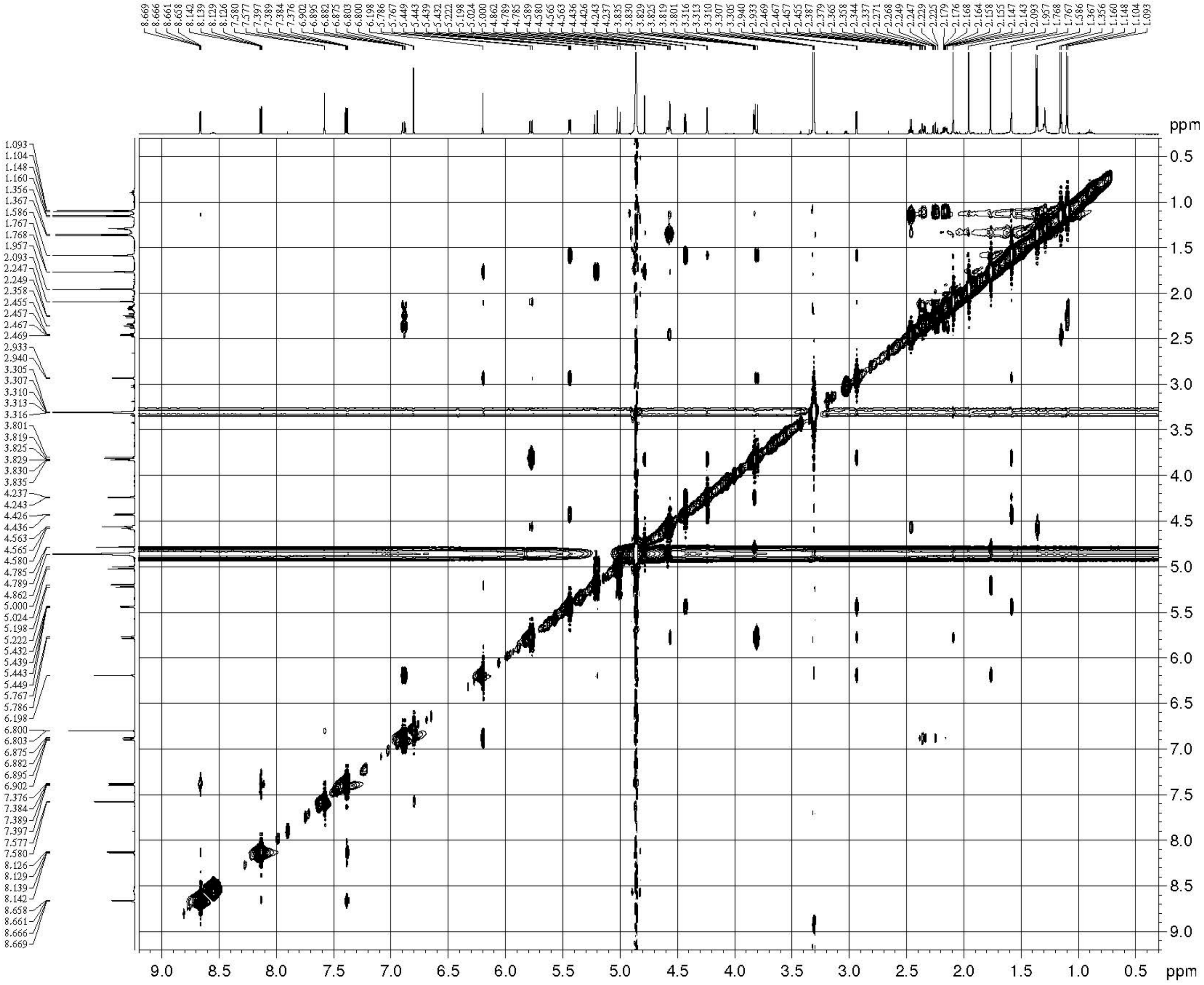

Figure S17. NOESY spectrum of compound **2** in CD_3OD.

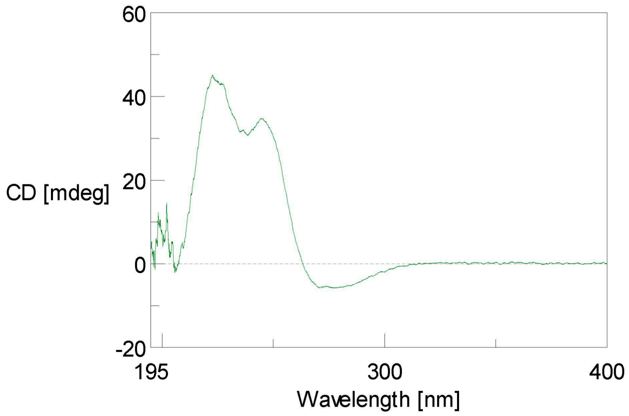

Figure S18. CD spectrum of compound **2** in MeOH.
